# Superfluous Hair: Some Methods of Treatment

**Published:** 1913-05-17

**Authors:** 


### SUPERFLUOUS HAIR.

### Some Methods of Treatment.

In an able and exhaustive survey of the subject of hypertrichosis, or excessive hairiness, Dr. D. Freshwater * discusses the relative merits and advantages of the different methods of circumventing this condition. He enumerates eight separate and distinct sorts of remedy, some of which are applied in different ways or can be subdivided into varying forms. And he also points out that, although as a rule it is the female sex which most often applies for relief from hypertrichosis, yet sometimes men also come for treatment of this condition.

The first method on the list is the plucking of individual hairs out by the roots, an unpleasant remedy, but highly effective for the time being. Unfortunately, the growth recurs, and may even be of a stronger sort than before. Moreover, if the number of offending hairs be large, considerable inflammation is excited by this process, which is on many grounds inferior to some of the less ancient devices.

Shaving, with which may be included cutting and singeing, is held in disfavour by ladies, owing to the widespread impression that shaving stimulates the hair-growth. It is, nevertheless, extremely valuable in many cases, and although it may be admitted that individual hairs seem to grow thicker after some years of shaving, yet there is no increase in the actual number of hairs. As a matter of fact, shaving is so thoroughly effective for the time being, so harmless, and so safe and painless, that the disfavour in which it is often held is somewhat surprising. By using a safety razor once a fortnight or so, many ladies are able to conceal quite successfully from the world at large that they are the subjects of hypertrichosis.

The *x*-rays, as is well known by now, will cause the hair to fall from any part which is exposed to a sufficient dose: this fact is taken advantage of in the treatment of ringworm. A somewhat larger dose causes permanent, instead of temporary, baldness, and a good many cases of the kind have occurred during the *x*-ray treatment of ringworm. Can the rays, therefore, be made use of to produce intentional destruction of the hair-roots? Dr. Freshwater is against it on the grounds that scars, pigmentation, or telangiectasis may be accidentally produced, and may be permanent. This would indeed be out of the frying-pan into the fire.

Next on the list stand depilatory drugs, which, like tweezer-plucking, have been used in the harems of the East for many centuries. These substances, when applied to the hairy part, soften and destroy that part of the hair shaft to which they get access. In other words, in order to destroy the hidden part of the hair, they would have to be left on long enough to eat away the skin itself, which would leave a scar. Depilatories, therefore, are no more effective than shaving, and have to be reapplied at much the same interval of time. Moreover, they tend to cause eventually a thicker growth, so that their advantage over shaving is much less real than apparent. No preparation, says Dr. Freshwater, has yet been discovered which will with certainty produce permanent removal of the hair without destroying also the skin. Although many advertised secret nostrums pretend to this, their extravagant claims are far in excess of their performances.

* *Practitioner*, May, 1913, p. 832.

Yet another method is that of bleaching the hair by means of hydrogen peroxide or some preparation containing it, thus rendering it comparatively inconspicuous. This is, naturally, most useful in those cases where the hairs are numerous but minute. Besides bleaching the hair, preparations of this kind after a while render it brittle and much more likely to fall, and they also retard to some degree its further growth. This method is therefore harmless and fairly effective for some cases, which is more than can be said of all the remedies for hypertrichosis.

The use of pumice-stone is also not to be despised as an auxiliary. After shaving the affected part, a piece of smooth pumice-stone is rubbed gently over the skin against the direction of the hair-growth twice a day. The friction must be quite gentle, and the place should be anointed with cold cream afterwards. This is done every day for six months, and then a rest of one month is taken. The new growth of hair will probably be found to be much weaker and more scanty, and a decision can then be taken whether another six months of the treatment is necessary. If this succeeds, an occasional rubbing every now and then will sustain the improvement.

Last come methods of epilation, which aim at destruction of the hair-root, so preventing any return of the growth. One drastic method for doing this is known by the name of "Kromeyer," its inventor, but the risks of this severe process are generally considered to contraindicate its use. The alternative is electrolysis, which is safe, effective, and not very uncomfortable. Unfortunately, it is so tedious that only twenty to thirty hairs can be dealt with at one sitting: also it is of much more value for thick sparse hairs than for a great number of fine downy ones. About 80 per cent. of the hairs thus dealt with never grow again, but the operator requires considerable experience to be able to get results as good as this. Various precautions are advisable if scarring is to be avoided, and it is well to pick out hairs at some distance from one another and uniformly distributed over the region to be depilated. It is also necessary to warn the patient that a skin which has shown a tendency to hypertrichosis may continue to do so, and that new hairs may appear which would have done so in any case, otherwise the treatment may be thought to have failed, and disappointment may ensue.

